# Yangxinkang tablet protects against cardiac dysfunction and remodelling after myocardial infarction in rats through inhibition of AMPK/mTOR-mediated autophagy

**DOI:** 10.1080/13880209.2020.1748662

**Published:** 2020-04-14

**Authors:** Pei-hua Ren, Zhi-min Zhang, Peng Wang, Han-ping Zhu, Zhen-qiu Li

**Affiliations:** Department of Traditional Chinese Medicine, The First Affiliated Hospital of Guangzhou Medical University, Guangzhou, China

**Keywords:** Acute myocardial infarction, heart injury, cardiac fibrosis, Chinese herbal compound

## Abstract

**Context:**

Acute myocardial infarction (AMI) is defined as myocardial necrosis. Clinicians use the traditional Chinese patent medicine Yangxinkang Tablet (YXK) to treat chronic heart failure.

**Objective:**

To explore the effects of YXK on heart injury following AMI and the underlying mechanisms.

**Materials and methods:**

The AMI model was produced in Wistar rats by permanent ligation of the left anterior descending coronary artery. Rats were divided into the following five groups: Sham (*n* = 6), MI (Model, *n* = 10), AICAR (AMPK agonist, 50 mg/kg/d, i.p., *n* = 10), Compound C (AMPK inhibitor, 10 mg/kg/d, i.p., *n* = 10), and YXK (0.72 g/kg/d, gavage, *n* = 10) groups. Cardiac function, cardiac fibrosis, apoptosis, and expression of p-AMPK, p-mTOR, and autophagy-related proteins was measured after 4 weeks of treatment after the successful modelling of the AMI.

**Results:**

Compared to MI group, both YXK and AMPK inhibitor improved cardiac dysfunction and reduced cardiac fibrosis (15.6 ± 2.3; 22.6 ± 4.6 vs. 34.6 ± 4.3%) and myocardial cell apoptosis (12 ± 3.67; 25.6 ± 6.8 vs. 54 ± 4.8%). Futhermore, YXK and AMPK inhibitor significantly decreased p-AMPK expression by 11.05% and 14.64%, LC3II/I by 25.08% and 35.28% and Beclin-1 by 66.71% and 33.85%, increased p-mTOR by 22.14% and 47.46% and p62 by 70.83% and 18.58%.

**Conclusions:**

The underlying mechanism appears to include suppression of autophagy via inhibiting AMPK/mTOR signalling, suggesting that YXK may serve as a potentially effective Chinese herbal compound for suppressing cardiac fibrosis in heart injury.

## Introduction

Acute myocardial infarction (AMI)-induced heart failure is one of the most frequently occurring heart diseases, and it contributes to high mortality in the world. Generally, myocardial infarction (MI) is the result of coronary arterial occlusion. MI leads to myocardial remodelling of the left ventricle, presenting as heart cavity dilatation, poor cardiac performance, arrhythmias, and even heart failure. A great number of factors are involved in cardiac remodelling post-MI, including myocardial cell death, apoptosis, and inflammation (Talman and Ruskoaho 2016). However, the process of pathological alteration of heart after MI is complicated, and it requires further study of novel targets and drug development for the treatment of MI.

Autophagy is a natural process where long-lived proteins and damaged organelles are degraded and recycled, resulting in the turnover of long-lived proteins and damaged organelles (Mizushima and Komatsu 2011). Autophagy includes the following three different processes: macroautophagy, microautophagy, and chaperone-mediated autophagy. In this study, we focus on macroautophagy, which is generally referred to as autophagy. A defect in the autophagic process can promote cell apoptosis and cell death (Thorburn 2008). Autophagy was reportedly enhanced by MI and exerted protective effects on cardiac fibrosis and cardiac function (Wu et al. 2014).

In contrast, emerging evidence suggests that autophagy is detrimental under certain circumstances. Researchers have found that excessive autophagy could promote cardiomyocyte death during reperfusion (Matsui et al. 2007), which likely occurred through the destroying of a large fraction of organelles (Zhu et al. 2007). Moreover, the suppression of autophagy could reduce MI sizes (Wang et al. 2015). Therefore, the role of autophagy in MI remains controversial. AMP-activated protein kinase (AMPK), a serine-threonine kinase, is important for maintaining energy homeostasis during cellular stress. AMPK activation can inhibit rapamycin (mTOR) via the mammalian target and, thus, triggers autophagy. AMPK-mTOR signalling plays a crucial role in cardiac function post-MI (Qi and Young 2015).

Chinese medicine is widely used in clinical treatments in countries of Southeast Asia, including chronic heart failure, angina, and MI. Yangxinkang tablet (YXK) is a Chinese herbal compound, primarily comprising of ginseng, astragalus, radix ophiopogonis, schisandra, and pubescent holly root. Our previous studies (Peihua Ren et al. 2018a, 2018b) showed that treatment with YXK improved cardiac function in rabbits post-MI. Here, we continue to study the effects of YXK on cardiac remodelling in a rat model post-MI and the relevant underlying mechanisms.

## Materials and methods

### MI model and experimental protocols

Animal experiments were approved by the Institutional Animal Care and Use Committee at The First Affiliated Hospital of Guangzhou Medical University, Guangzhou, China. The animals were treated in accordance with the Guide for the Care and Use of Laboratory Animals (8th edition, National Academies Press). Wistar rats (250 g, 7–8 weeks old) were obtained from Medical Experimental Animal Centre of Guangdong Province, Guangzhou, China. The MI model was created in accordance with a modified method in previous study (Wu et al. 2011). Briefly, animals were anesthetized (by 5 mL/kg of 1% pentobarbital, i.p.) and artificially ventilated using a respirator. Next, the thorax was opened at the left third intercostal space, and MI was induced by ligating the proximal left anterior descending (LAD) coronary artery. The successful infarction was identified by visually observing a change of the colour of the anterior wall of the left ventricle from red to blanching and cyanosis and swelling of the left atrium. Animals in the sham group were subjected to the same surgical procedure but excluding the ligation LAD coronary artery.

Rats were randomly assigned to five groups as follows: sham group (Sham, *n* = 6); MI group, MI rats administered saline alone (*n* = 10); AICAR group (AMPK agonist), MI rats treated with AICAR (50 mg/kg, i.p., *n* = 10) (Robert et al. 2009); Com C group, MI rats treated with Compound C (10 mg/kg, i.p., *n* = 10) (Abdulrahman et al. 2014); YXK group, MI rats treated with YXK solution (0.72 g/kg/d, gavage, *n* = 10). All the treatments were provided one day after the induction of MI. They were provided once a day and continued for four weeks post-MI. AICAR and Compound C were purchased from Aladdin Company, Shanghai, China. YXK tablet was provided by The First Affiliated Hospital, Guangzhou University of Traditional Chinese Medicine, China, and the dose of YXK used in this study was twice the clinical equivalent dose. YXK solution was freshly prepared with saline for each daily experiment.

### Echocardiography

Transthoracic echocardiography was performed using colour ultrasound diagnosis system (SonixTOUCH, Ultrasonix Medical Corp., Canada) equipped with 1–5 MHz probe. Rats were anesthetized with 2% isoflurane gas during the examination. The structure and function of the left ventricle were evaluated using long axis view and short axis view. Chamber diameters (left ventricular [LV] internal diameter at end-diastole and end-systolic) and LV fractional shortening (LVFS) were measured by M-mode. LV ejection fraction (LVEF) was calculated from the apical four-chamber view.

### Masson’s staining

After four weeks of treatment as described above, rats were anesthetized and sacrificed. The hearts were perfused, fixed with 10% buffered formalin, and cut into 5 µm sections, followed by Masson’s staining according to the manufacturer’s instructions (Servicebio Company, Wuhan, China).

### TUNEL assay

Myocardial cell apoptosis was determined using the DeadEnd™ Fluorometric TUNEL System (Promega, USA). Staining using TUNEL assay was performed following the manufacturer’s instructions. Briefly, fixed heart tissue slices were permeabilized in 0.2% Triton X-100 in PBS for 5 min. Next, the slices were equilibrated at room temperature for 10 min and incubated with TdT reaction mix for 60 min at 37 °C in a humidified chamber. Lastly, Hoechst 33258 (Invitrogen, USA) was used to label nuclei. Next, the slices were mounted. The images were captured using fluorescence microscopy. The apoptotic cells showed as green fluorescence and nuclei showed as blue. The apoptosis percentage was calculated by counting at least six randomly taken visual fields.

### Western blotting

Heart tissues were lysed with RIPA and centrifuged at 12,000 rpm for 20 min. The protein concentration in supernatants was determined using a Bradford kit (Pierce, USA). Protein samples (50 μg) were loaded and separated using SDS–PAGE gels (10–15%). Subsequently, we transferred the protein to PVDF membranes (Millipore, Bedford, MA, USA). The membranes were blocked and incubated with primary antibodies against Bax (Cell Signalling, USA), Bcl2 (Abcam, USA), p-AMPKα (Cell Signalling, USA), AMPK (Cell Signalling, Danvers, MA, USA), LC3 (Cell Signalling, USA), Beclin-1 (Cell Signalling, USA), SQSTM1/p62 (Cell Signalling, USA), mTOR (Abcam, USA), p-mTOR (Abcam, USA), and GAPDH (TransGen Biotech, China) at dilutions recommended by the antibody datasheet. After the samples were washed three times with TBST, the membranes were incubated with the corresponding HRP-secondary antibody diluted at 1:3000 (Forevergen Company, China). The blots were developed using Pluslight ECL Kit (Forevergen Company, China). The densitometry analysis of bands was performed using Image J software.

### Real-time PCR

Total RNA was isolated using Trizol (Molecular Research Centre, Inc., USA). A total of 2 µg RNA was used to synthesize cDNA using M-MLV Reverse Transcriptase (Promega, USA). The quantitative PCR reaction system included 1.5 µL cDNA, 0.5 µL of each primer (10 μM), and 10 µL ChamQ SYBR qPCR Master Mix (Vazyme Biotech Co., Ltd, China). The PCR reaction was performed in the StepOne system (Applied Biosystems, StepOne Plus, USA). The sequences of the primer pairs are as follows: Beclin-1, 5′-CTCTCGCAGATTCATCCCCC-3′/5′-CTCCCCGATCAGAGTGAAGC-3′; LC3, 5′-CATGAGCGAGTTGGTCAAGAT-3′/5′-TCGTCTTTCTCCTGCTCGTAG-3′; p62/SQSTM1, 5′-TGTGGAACATGGAGGGAAGAG-3′/5′-TGTGCCTGTGCTGGAACTTTC-3′; and GAPDH, 5′-ATCAAGTGGGGTGATGCTGG-3′/5′-CCTGCTTCACCACCTTCTTGA-3′. The relative expressions of LC3, Beclin-1, and p62 were normalized to GAPDH.

### Statistical analysis

Results are presented as mean ± SD. For comparison of more than two groups, one-way analysis of variance was performed. We used GraphPad Prism 7.0 (Graphpad Software Inc., La Jolla, CA, USA) and SPSS software (IBM, USA) for the statistical analysis. *p* < 0.05 was considered significant.

## Results

### YXK improved cardiac function post-MI

**Figure 1.**
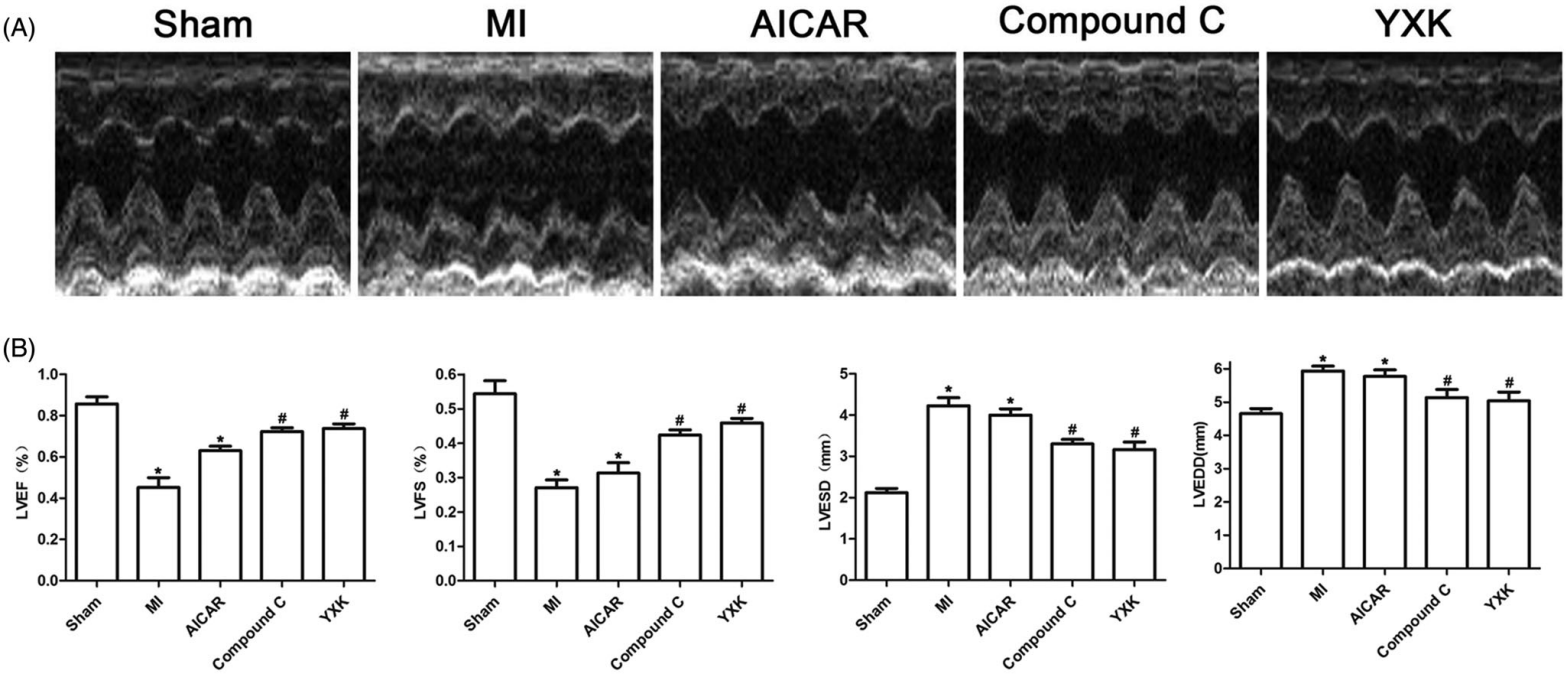
YXK and AMPK inhibitor Compound C improved cardiac performance after MI. Cardiac function was evaluated using echocardiography in rats by the treatment with YXK, AICAR, and Compound C for four weeks post-MI. (A) the representative images of echocardiographic assessment. (B) the analysis results of cardiac function, including LVEF, LVFS, LVESD, and LVEDD. *Versus sham group; #versus MI group; *p* < 0.05; sham group (*n* = 6), the other groups (*n* = 10).

First, we evaluated the effects of YXK on cardiac performance post-MI. Cardiac function was determined by echocardiography at four weeks post-AMI. As shown in Figure 1, when compared with the sham group, the MI group showed significant decreases in LV ejection fraction (LVEF) (0.45 ± 0.07 vs. 0.86 ± 0.05%), LV fractional shortening (LVFS) (0.27 ± 0.03 vs. 0.54 ± 0.05%), and increases in the LV end-systolic dimension (LVESD) (4.33 ± 0.14 vs. 2.12 ± 0.15 mm) and LV end-diastolic dimension (LVEDD) (5.9 ± 0.2 vs. 4.65 ± 0.22 mm). Both YXK and the APMK inhibitor Compound C treatment for four weeks significantly improved cardiac function showing as significant increases in LVEF (0.77 ± 0.03 and 0.65 ± 0.02 vs. 0.45 ± 0.07%) and LVFS (0.46 ± 0.03 and 0.37 ± 0.01 vs. 0.27 ± 0.03%), but decreases of LVEDD (5.34 ± 0.05 and 5.42 ± 0.21 vs. 5.9 ± 0.2 mm) and LVESD (2.87 ± 0.17 and 3.4 ± 0.21 vs. 4.33 ± 0.14 mm) compared with the MI group. However, the AMPK activator AICAR treatment did not show obvious effects on the cardiac function compared with the MI group.

### YXK attenuated cardiac remodelling and myocardial cell apoptosis after MI

**Figure 2.**
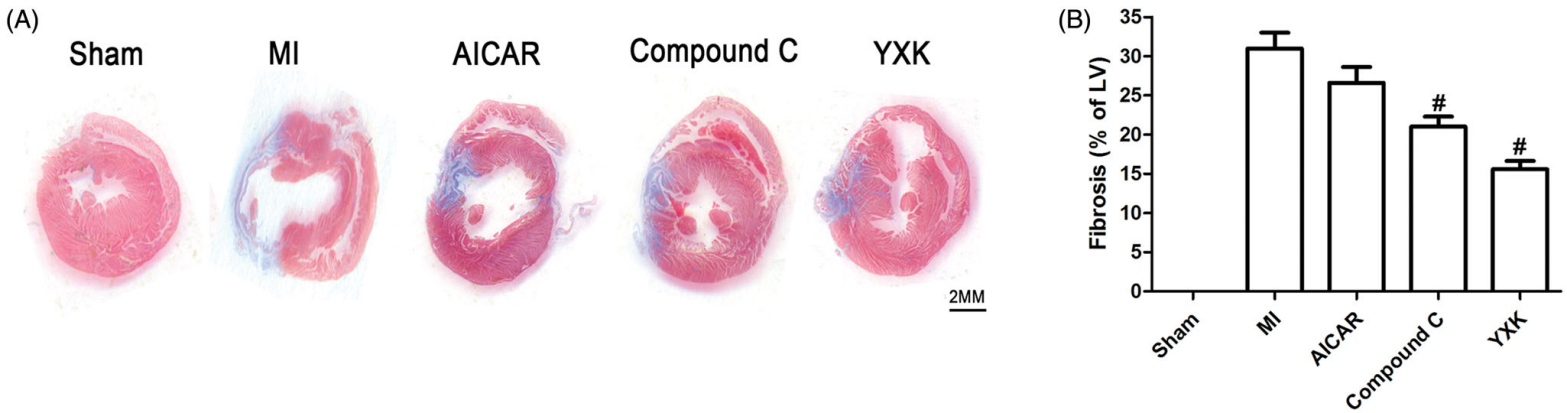
YXK and AMPK inhibitor Compound C reduced cardiac fibrosis of hearts in each experimental group was collected and sectioned at the indicated time-point. (A) Masson’s staining was used to determine myocardial fibrosis after MI. The tissues stained with blue colour represented fibrosis. (B) The percentage of fibrosis area was compared among groups. #Versus MI group, *p* < 0.05; sham group (*n* = 6), the other groups (*n* = 10).

We performed Masson staining to study the effects of YXK and AMPK activator/inhibitor on cardiac fibrosis post-infarction. As shown in Figure 2(A,B), heart tissues in the MI group showed increased fibrosis and scar formation (blue staining), while these findings were reduced in YXK and APMK inhibitor Compound C treatment (15.6 ± 2.3 and 22.6 ± 4.6 vs. 34.6 ± 4.3%). AMPK activator AICAR did not show significant effect on cardiac fibrosis in MI rats.

**Figure 3.**
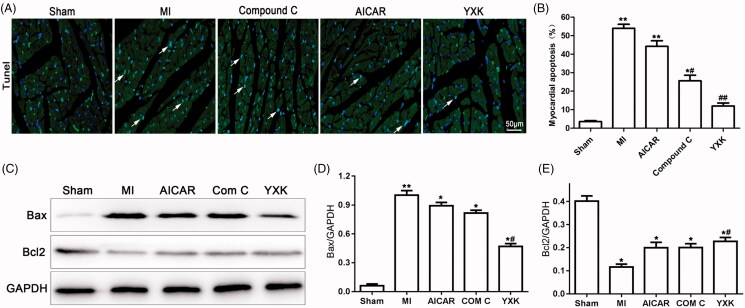
YXK and AMPK inhibitor Compound C reduced myocardial apoptosis after MI. Hearts in each experimental group was collected and sectioned at the indicated time-point. Staining using TUNEL assay was performed to determine cell apoptosis. (A) the representative images of TUNEL-stained cells were shown. The arrows indicate the apoptotic cells. (B) The apoptotic percentages were compared between groups. (C–E) the expressions of Bax and Bcl2 protein were determined by western blotting. (**versus sham group, *p* < 0.01; #versus the MI group, *p* < 0.05; ##versus the MI group, *p* < 0.01; sham group *n* = 6, the other groups *n* = 10).

Staining using TUNEL assay was performed to determine myocardial cell apoptosis post-MI. As shown in Figure 3(A,B), the percentage of TUNEL-positive myocardial cells significantly increased post-MI compared with sham group, suggesting MI-induced myocardial cell apoptosis, which attributed to poor cardiac performance. Both YXK and APMK inhibitor Compound C treatment significantly reduced cardiac apoptosis post-MI compared with MI group (12 ± 3.67 and 25.6 ± 6.8 vs. 54 ± 4.8%). Moreover, the YXK treatment group displayed lower cardiac apoptotic rate than that of Compound C treatment group. AMPK activator AICAR did not affect cardiac apoptosis compared with the MI group in a distinct pattern. To further confirm the apoptotic effect of the YXK, we used western blotting to detect the expression of the apoptosis proteins Bax and Bcl2.

Figure 3(C,D) results showed that Bax protein expression in heart tissues was significantly increased. Bcl2 was decreased in the MI group when compared with sham group. The patterns for these two proteins were reversed by YXK treatment and Compound C treatment groups.

### YXK suppressed AMPK/mTOR signalling pathway

**Figure 4.**
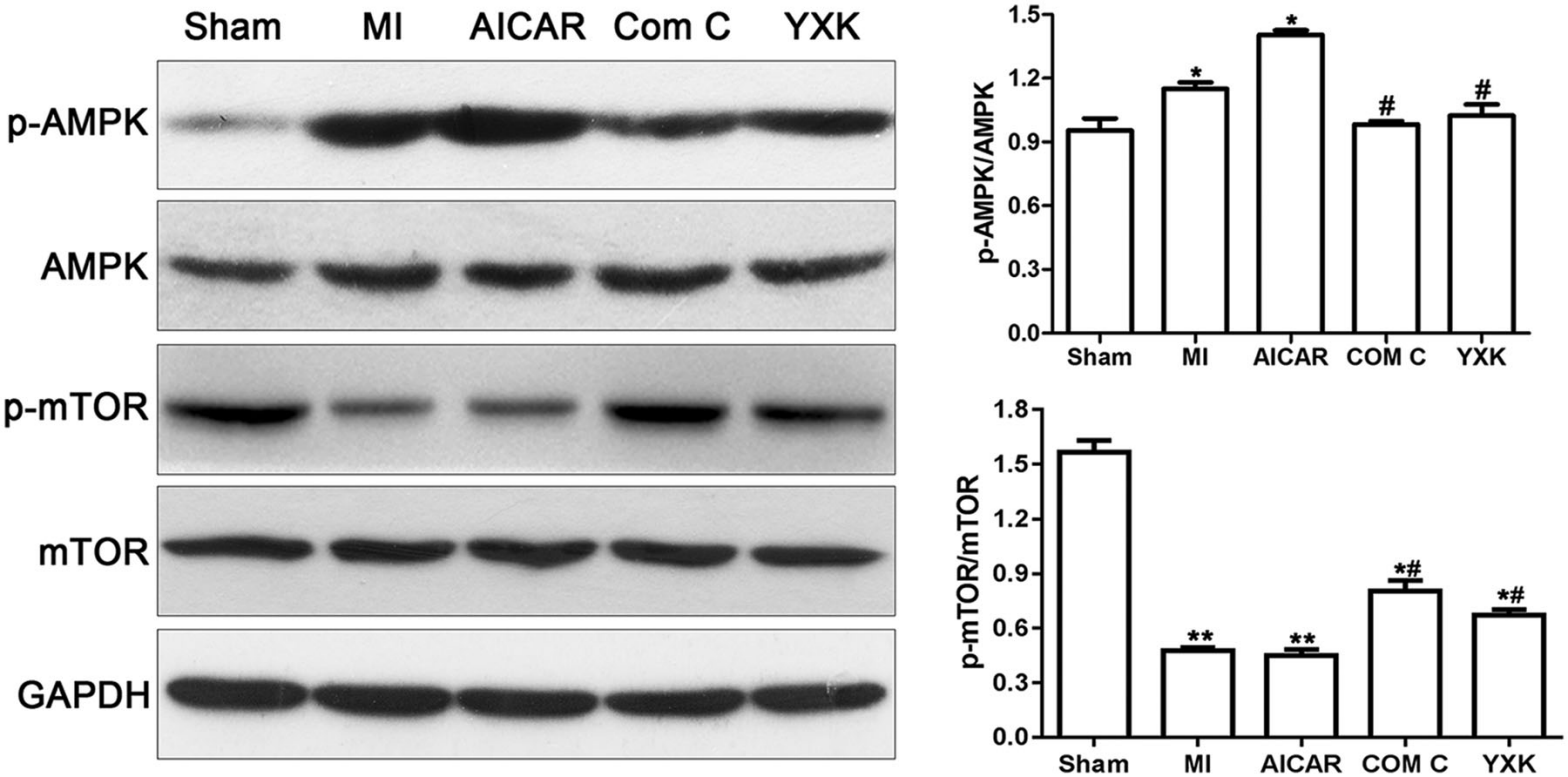
YXK and AMPK inhibitor Compound C inhibited AMPK-mTOR signalling in rat heat after MI. After treatment with YXK, AICAR, and Compound C (Com C) for four weeks post-MI, heart tissues were collected and AMPK-mTOR signalling-related proteins were determined using western blotting. The representative images of western blot were shown (sham group *n* = 6, the other groups *n* = 10).

We investigated the underlying signalling pathways involved in the protective effect of YXK on cardiac dysfunction induced by MI. As shown in Figure 4, western blotting showed a significant increase in the phosphorylation of AMPK (p-AMPK) but decrease in the phosphorylation of mTOR (p-mTOR), indicating that the MI activated the AMPK-mTOR signalling pathway. YXK treatment significantly reduced the p-AMPK and increased the p-mTOR expression as well. APMK inhibitor Compound C also showed similar effects as YXK. The expression of the total AMPK and mTOR protein was not significantly different between groups.

### YXK inhibited MI-induced cardiac autophagic flux disorder

**Figure 5.**
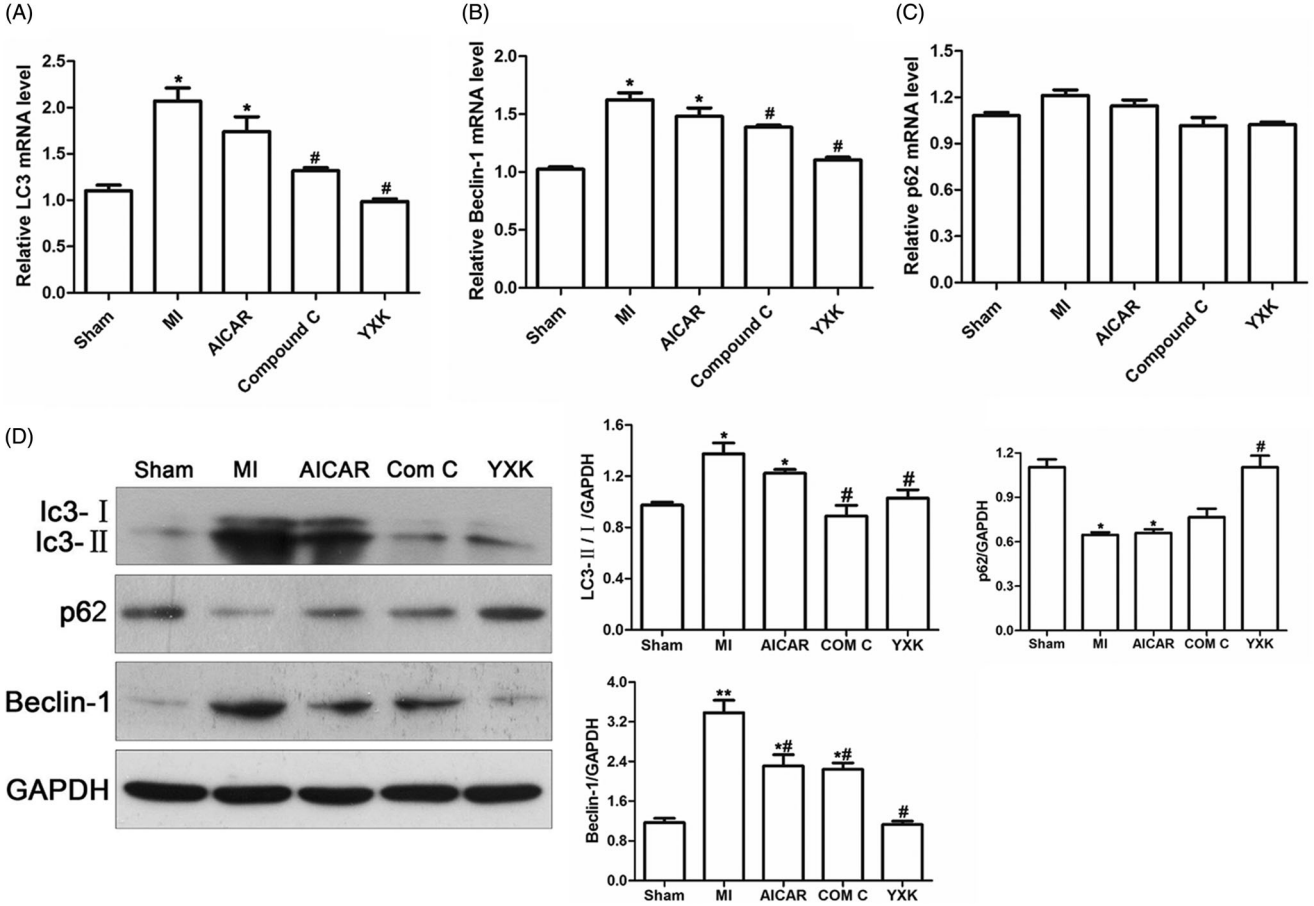
YXK and AMPK inhibitor Compound C improved autophagic flux in rat heart after MI. After treatment with YXK, AICAR, and Compound C (Com C) for four weeks post-MI, heart tissues were collected to determine the mRNA and protein expression of LC3, Beclin-1, and p62 using qPCR and western blotting, respectively. (A–C) qPCR results of LC3, Beclin-1, and p62 (*versus sham group, #versus the MI group, *p* < 0.05, *n* = 6–10). (D) Western blotting results of LC3II/LC3I, Beclin-1, and p62. The representative images were shown (sham group *n* = 6, the other groups *n* = 10).

Western blotting and real-time PCR were performed to determine the expression of autophagy-related protein expression. As shown in Figure 5(A,B), compared with sham group, MI significantly induced increased expression of LC3 and Beclin-1 mRNA, both of which were significantly reduced by YXK treatment. AMPK inhibitor Compound C also inhibited these gene expressions. No significant difference was noted in LC3 and Beclin-1 gene expressions between MI group and AICAR treatment group. The expression of p62 mRNA was not significantly different in these groups (Figure 5(C)). Western blotting results showed that the ratio of LC3II/LC3I and Beclin-1 protein expression increased in MI group compared with sham group, and they were decreased by YXK and Compound C treatment (Figure 5(D)). AICAR treatment significantly reduced the Beclin-1 protein expression comparing with MI group. As shown in Figure 5(D), p62 protein expression in heart tissues was significantly decreased in MI group compared with sham group, and it was restored by treatment with YXK. Both AMPK activator AICAR and AMPK inhibitor compound C had no apparent effect on the protein expression compared with the MI group.

## Discussion

MI, also termed heart attack, is caused by the decrease in or stopping of blood supply to a part of the heart, and it leads to cardiac fibrosis, inferior cardiac function, and, ultimately, heart failure (Pouleur et al. 2010; Kanamori et al. 2011; Wu et al. 2014). The change in cardiac function is seen as a decrease of cardiac output, fractional shortening and ejection fraction, and an increase in LV end-systolic and LV end-diastolic dimension. Reportedly, the cardiac function was significantly reduced in mice after MI at 7- and 21-days compared with that in the sham group (Wu et al. 2014). Meanwhile, cardiac fibrosis was also found in the heart of mice at 7- and 21-days post-MI (Wu et al. 2014). The MI produced in rats and rabbits showed results similar to those in mice (Chi et al. 2017; Yang et al. 2018), In this study, we evaluated cardiac function and fibrosis for four weeks post-MI using echocardiography and Masson’s staining, respectively. In line with the previous studies, we found poor cardiac performance four weeks after MI. YXK treatment (gavage) for four weeks significantly improved both cardiac function and cardiac fibrosis in rats with MI. These findings provide the experimental evidence that YXK is an effective Chinese herbal compound for the treatment of heart failure after MI.

Generally, myocardial cells lack proliferative ability. Myocardial cell death or apoptosis resulted from MI insult is a significant cause for impaired cardiac function and cardiac fibrosis (Kajstura et al. 1996; Kurrelmeyer et al. 2000). In our study, the results showed that MI induced increased myocardial cell apoptosis accompanied with an increase in Bax protein expression and a reduction in Bcl2 protein expression, YXK treatment significantly inhibited myocardial cell apoptosis after MI, suggesting that the improvement of cardiac function by YXK was at least partially due to reduced myocardial cell apoptosis.

Autophagy is a conserved activity controlling protein degradation and the clearance of damaged organelles. Regarding the autophagy-related signalling, LC3II is the marker for the formation of autophagosome, and the level of p62 protein reflects the activity of autophagic flux (Jiang and Mizushima 2015). Beclin-1 is also a critical molecular participating in autophagy, and it increased in heart after MI (Wu et al. 2014). When the cells are exposed to stressful stimuli, such as hypoxia and nutrient deprivation, autophagy will increase and act as a protective mechanism (Mizushima and Komatsu 2011). Autophagy was strengthened by MI and demonstrated protective effects on cardiac fibrosis and function (Wu et al. 2014). However, some evidence also showed that autophagy is detrimental to heart function under certain circumstances. For instance, excessive autophagy, mediated, by Beclin-1 aggravated cardiomyocyte death during reperfusion (Matsui et al. 2007), likely through destroying a significant fraction of organelles (Zhu et al. 2007). Moreover, suppression of autophagy could reduce MI sizes (Wang et al. 2015). In our study, we found that the ratio of LC3II/LC3I and Beclin-1 in the heart was increased after MI, while p62 protein expression was decreased after MI at four weeks, suggesting autophagy was enhanced in heart affected by MI. These results are consistent with other researchers’ reports (Wu et al. 2014). YXK treatment significantly suppressed the ratio of LC3II/I and beclin-1 protein expression, while it upregulated p62 protein expression in hearts after MI. This observation demonstrated that YXK inhibited autophagy induced by MI in heart. Taken together with the protective effect of YXK on rat heart after MI, these results indicated that YXK protected against MI-induced heart dysfunction, which might be induced through the abrogation of excessive autophagy by reducing LC3II and beclin-1 expression.

During the MI, there is a decrease in ATP concentration and the increase of AMP/ATP ratio, which ultimately leads to activation of AMPK. AMPK activation inhibits mTOR activity and, thus, can trigger autophagy in heart during MI. Several studies suggested that AMPK activation is beneficial for MI (Russell et al. 2004; Maiuri et al. 2007), enhancing AMPK-dependent autophagy improved heart injury induced by MI (Gonon et al. 2008). However, an early study also showed that AMPK activation was detrimental to ischaemic heart by stimulating fatty oxidation and subsequently reducing glucose oxidation (Zeng et al. 2018).

Moreover, the pro-apoptotic effects of AMPK activation in heart cells have been reported (Buss et al. 2009). Therefore, the activation of AMPK in MI may either be beneficial or harmful, depending on the stage of disease process and the metabolic circumstance. The role of AMPK/mTOR mediating autophagy in MI remains to be fully understood (Lassaletta et al. 2014). Our results showed that YXK significantly inhibited AMPK/mTOR signalling by reducing the level of p-AMPK and increasing the level of p-mTOR. In the meantime, our results showed that inhibition of AMPK/mTOR signalling using AMPK inhibitor Compound C, along with the decrease of p-AMPK expression and the increase of p-mTOR, significantly improved rat heart function after MI. Moreover, inhibition of AMPK by Compound C as well as YXK treatment was found to reduce the ratio of LC3II/LC3I and Beclin-1 protein expression, while there was increased p62 protein expression in the rat heart after MI in our study. Therefore, our findings indicated that inhibition of AMPK/mTOR signalling pathway attenuated autophagy induced by MI, which involved in the protective effects of YXK against heart dysfunction and cardiac fibrosis after MI.

## Conclusions

This study demonstrated that YXK protected against injury after myocardial infarction by inhibiting excessive autophagy induced by MI. The underlying mechanism likely involves the inhibition of AMPK/mTOR signalling pathway. Our data suggest that YXK may be a promising candidate for AMI therapy.
